# Practical Treatise on Nasal Catarrh

**Published:** 1885-11

**Authors:** 


A Practical Treatise on Nasal Catarrh. By Beverly Robinson, A. M., M. D. (Paris), Clinical Professor of Medicine at the Bellevue Hospital Medical College, New York, etc., etc. One hundred and fifty-two engravings. New York: Wm. Wood & Co. 1885.

The first edition of Dr. Beverly Robinson's book on Nasal Catarrh was a valuable contribution to medical literature, but the value of the work has been greatly enhanced by new chapters added to the second edition.

Every physician who has had much to do with nasal troubles knows that a large number of cases are complicated by diseases of the ear as a direct result of the inflammation of the nose. A new chapter in the second edition on this subject will demonstrate to any one the danger to good hearing in neglecting even slight nasal diseases.

Another new chapter on mucous nasal polypi adds much to the value of the book. A patient with nasal polypus said to me a few days ago: "Why has this disease increased so in frequency? I hear of cases in every community." They are, perhaps, not more numerous than formerly, but better modes of examination and more careful investigations discover polypus now which heretofore remained undiscovered.

The therapeutics of the book throughout are good, and will repay the close study of every physician interested in the treatment of nasal diseases. A. W. C.

